# Unraveling the Regulatory G-Quadruplex Puzzle: Lessons From Genome and Transcriptome-Wide Studies

**DOI:** 10.3389/fgene.2019.01002

**Published:** 2019-10-18

**Authors:** Subramaniyam Ravichandran, Jin-Hyun Ahn, Kyeong Kyu Kim

**Affiliations:** ^1^Department of Molecular Cell Biology, Institute for Antimicrobial Resistance Research and Therapeutics, Sungkyunkwan University School of Medicine, Suwon, South Korea; ^2^Samsung Biomedical Research Institute, Samsung Advanced Institute for Health Sciences and Technology, Samsung Medical Center, Sungkyunkwan University School of Medicine, Seoul, South Korea

**Keywords:** G-quadruplex, secondary structure, genome-wide study, transcriptome, transcription, gene expression

## Abstract

G-quadruplexes (G4s) are among the best-characterized DNA secondary structures and are enriched in regulatory regions, especially promoters, of several prokaryote and eukaryote genomes, indicating a possible role in *cis* regulation of genes. Many studies have focused on evaluating the impact of specific G4-forming sequences in the promoter regions of genes. However, the lack of correlation between the presence of G4s and the functional impact on *cis* gene regulation, evidenced by the variable expression fold change in the presence of G4 stabilizers, shows that not all G4s affect transcription in the same manner. This indicates that the regulatory effect of the G4 is significantly influenced by its position, the surrounding DNA topology, and other environmental factors within the cell. In this review, we compare individual gene studies with high-throughput differential expression studies to highlight the importance of formulating a combined approach that can be applied in humans, bacteria, and viruses to better understand the effect of G4-mediated gene regulation.

## Introduction

The landscape of genomic DNA has shown a myriad of alternate DNA structures such as cruciform (Brazda et al., 2011), G-quadruplexes (G4s) (Kwok and Merrick, 2017), triplexes (Frank-Kamenetskii and Mirkin, 1995), and i-motifs (Abou Assi et al., 2018). These structures can form within genomic DNA (B-DNA), as seen in the case of left-handed Z-DNA (Kim et al., 2009), or require the opening of base pairs leading to generation of single-stranded regions within genomic DNA as seen in cruciform DNA (Brazda et al., 2011) and G4s (Kreig et al., 2015). Genome-wide prediction of secondary structure-forming regions in various genomes is possible because of their propensity to favor specific sequence patterns. It has been proven that Z-DNA favors purine–pyrimidine repeats flanked by specific sequences for B-DNA/Z-DNA junction formation (Bothe et al., 2011; Kim et al., 2018), whereas cruciform structures can be formed in palindromic regions (Leach, 1994). The abundance of secondary structures has led to attempts to identify the probable roles of these structures in replication, gene regulation, and DNA damage/repair. These structures have been implicated in several diseases, such as amyotrophic lateral sclerosis, frontotemporal dementia, Fanconi anemia, Bloom’s syndrome, and fragile X disease (Wu and Brosh, 2010; Simone et al., 2015).

G4s are among the most widely studied DNA secondary structures formed from consecutive blocks of two or more guanines separated by a single-stranded region called a loop. Four consecutive G-runs form G-stacks with Hoogsteen bonds [(C2)NH2:N7 and O6:N1H], which are stabilized by several monovalent and divalent cations such as K^+^, Na^+^, Ca^2+^, and Sr^2+^, which have been reviewed elsewhere (Sannohe and Sugiyama, 2010; Bhattacharyya et al., 2016). K^+^ is the best stabilizer of the G4 due to its favorable ionic radius and Gibbs free energy of solvation (Zaccaria et al., 2016). Computational tools such as Quadparser, PQSFinder, G4Hunter, and QGRS Mapper have been developed to predict putative G4-forming sequences (Parveen et al., 2019). They are based on pattern matching and scoring algorithms using the schema G_x_N_y_G_x_N_y_G_x_N_y_G_x_, where N is any nucleotide, x is ≥2, and y is ≥1. However, y is usually considered as 1–7 as longer loops are flexible and destabilize the G4 (Hazel et al., 2004). Several putative G4-forming sequences were predicted in the genomes of prokaryotes and eukaryotes and accessible through web servers such as QuadBase and NonBDB (Yadav et al., 2008; Cer et al., 2011). In addition, high-throughput sequencing has also been used to experimentally verify G4 formation in the genomes of many organisms (Marsico et al., 2019) and construct whole-genome experimental G4 maps.

**Figure 1:**
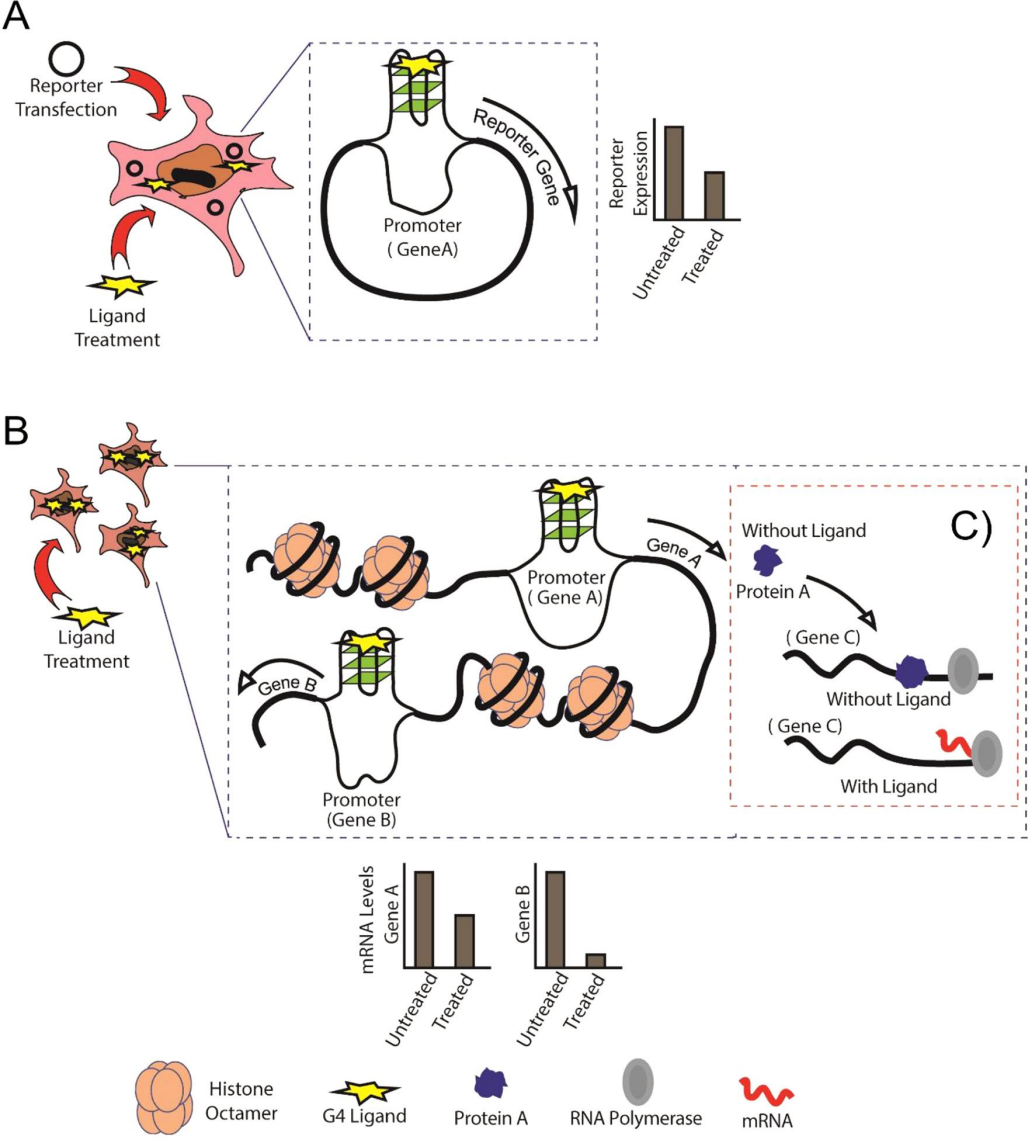
Schematic showing the comparison between conventional reporter assays and Genome-Wide Association Studies (GWAS). **(A)** Conventional reporter-based study of G-quadruplexes (G4s) in the specific gene promoters, in this case, Gene A. **(B)** GWAS expression analysis showing binding of G4 ligand to G4s formed in untargeted regions (Gene B) of the genome. **(C)** Protein A is normally expressed and performs its regulatory activity on Gene C in the absence of G4 ligand, whereas G4-mediated cis-regulation in the presence of G4 ligand leads abnormal regulation of Gene C by Protein A.

The enrichment of G4s throughout several genomes especially in the *cis*-regulatory regions (Chambers et al., 2015; Marsico et al., 2019) has led to the development of several small molecules that can bind and stabilize G4s, including porphyrins, benzoquinolines, and perylene diimide (Tian et al., 2018). Some of the most widely used G4 stabilizers are TMPyP4, NMM-IX, pyridostatin (PDS), and BRACO-19. To study the effects of these chemicals on G4-mediated gene regulation, individual reporter assays are performed by cloning specific regulatory regions in reporter vectors and analyzing reporter gene expression in the presence of a G4-stabilizing ligand (Halder et al., 2012a) (**Figure 1**). G4-mediated *cis*-regulatory activity has been confirmed by reporter assays in various genes such as *CMYC*, *C-KIT*, and *BCL2* (Siddiqui-Jain et al., 2002; Ashman and Griffith, 2013; Le et al., 2013).

Although these ligands display considerable selectivity for G4 structures over single-stranded and double-stranded DNA, it is essential to study the effect of ligand binding to untargeted G4-forming regions in the genome (**Figure 1**). A key factor influencing specificity and the *cis*-regulatory impact is to identify the conditions that affect G4 formation. Apart from intracellular K^+^, Na^+^, and Mg^2+^, there are several other conditions that can affect G4 formation within both prokaryotic and eukaryotic cells. Proteins that can interact with G4s have been described (Brazda et al., 2014; Hale et al., 2014). Some studies have shown that G4 formation is influenced by the chromatin status and that euchromatin shows more G4-forming sequences than heterochromatin (Hansel-Hertsch et al., 2016), corroborating the idea that actively transcribed genes show higher propensity to form G4s. Several universal transcription factors, such as MYC, SP1, and VEGF, are also regulated by G4s. Changes in expression levels of such transcription factors may affect the expression of genes regulated by them (**Figure 1**).

The cross-reactivity between G4 ligands and i-motifs has also been reported since i-motifs are present on the opposite strand of the G4. Ligands such as TMPyP4 and berberine were shown to bind to i-motifs, although their ability to stabilize them was lower than that of G4s (Fedoroff et al., 2000; Masoud and Nagasawa, 2018; Pagano et al., 2018). The prevalence of G4s in the RNA has also been shown recently (Kwok et al., 2016; Yang et al., 2018), and the functional effects of RNA G4 stabilization have also been reviewed before (Fay et al., 2017). DNA G4-binding ligands such as TMPyP4 have also been shown to destabilize RNA G4s (Ofer et al., 2009; Morris et al., 2012; Zamiri et al., 2014). However, the functional impact of the cross-reactivity of G4 ligands to DNA and RNA has not been well studied. A combination of high-throughput studies along with individual RNA analysis can also be utilized to understand the impact of cross-reactivity of G4 ligands. In addition, since RNA G4s have been extensively discussed in previous reviews (Fay et al., 2017), we do not include the studies on RNA G4s in this review.

Therefore, the addition of a G4-stabilizing ligand can be expected to impact multiple regions in the genome and affect transcription of multiple genes at the same time. High-throughput studies do not provide a fine-grained analysis of the dynamics of individual genes regulated by G4s. Therefore, individual reporter assays are required to analyze the effect of G4s on specific target genes (**Figure 1**). Concomitantly, individual reporter assays on all genes of an organism combined with genome or transcriptome-wide studies can provide a better understanding of how individual G4s can regulate gene expression (**Figures 1A, B**). In this review, we discuss the importance of combining high-throughput experiments with studies on individual G4s in humans, bacteria, and viruses to obtain a better picture of G4-mediated *cis* regulation.

## Current Status of Studies on *cis*-Regulation by G4s

### Studies on *Cis*-Regulatory G4s in Humans

Quadparser-based computational analysis of the human genome for the prediction of G4-forming sequences based on the schema G_3+_N_1–7_G_3+_N_1–7_G_3+_N_1–7_G_3+_ initially revealed 370,000 G4 sequences (Huppert and Balasubramanian, 2005). Regulatory regions of the human genome were enriched in G4s (Huppert et al., 2008; Verma et al., 2008), and the distribution of G4-forming sequences was also dependent on the function of the gene; for example, tumor suppressors contained lower G4-forming sequences than did proto-oncogenes (Eddy and Maizels, 2006). Later studies showed that promoter G4 regions overlapped with DNAse hypersensitive sites in over 40% of human genes (Huppert and Balasubramanian, 2007). Experimental confirmation of the impact of G4 formation on transcription and translation was carried out initially in specific genes such as *CMYC* (Yang et al., 2017), *KRAS* (Cogoi and Xodo, 2006), *HRAS* (Membrino et al., 2011), and *BCL2* (Nagesh et al., 2010). However, experimental evidence of G4 formation to corroborate the computational analysis was still pending. Later, high-throughput sequencing studies *in vitro* showed that over 700,000 G4s can be formed in the genome in the presence of KCl and PDS (Chambers et al., 2015). These studies proposed that G4 formation in the regulatory regions may have an impact on gene regulation. However, they could not elucidate the impact of nonspecific binding of G4 stabilizers or the downstream impact of G4-mediated gene regulation on other genes. These limitations may be overcome by high-throughput transcriptome-wide differential expression studies.

Several studies have involved treating cells for specific periods of time with G4-stabilizing ligands and analyzing changes in gene expression for changes before and after treatment (**Table 1**). In most studies, TMPyP4 was used as the G4-stabilizing ligand. Initial studies using the HeLa S3 cell line showed that the G4-binding ligand could cause changes in gene expression (Grand et al., 2002). They observed that proto-oncogenes, such as *CMYC*, *CMYB*, and *CFOS*, were downregulated under TMPyP4 treatment, but not by TMPyP2. Another study on the same cell line showed similar results and found that the promoter regions of differentially expressed genes, including *CMYC*, *CMYB*, and *CFOS*, contained G4-forming sequences (Verma et al., 2008). Interestingly, this study also found that there was no statistically significant correlation between the presence of G4s and the expression fold change. The same group performed a subsequent study with TMPyP4 on the A549 cell line and compared the results with BMVC and a TMPyP4 analog TyPy (Verma et al., 2009). This study also observed 863 significantly upregulated and 298 significantly downregulated genes similar to their previous study on Hela S3 cells. Therefore, they shortlisted 12 genes containing G4s in their promoters from the microarray results and analyzed them individually by quantitative Real-Time PCR (qRT-PCR), demonstrating that the genes were indeed affected by G4-stabilizing ligands.

**Table 1:** Compilation of studies on *cis*-regulatory G4s in humans, bacteria, and viruses.

List of high-throughput G4 cis-regulatory studies in *Homo sapiens*							
Organism	Computational prediction	Prediction algorithm	Ligand	Concentration of chemical used	Duration	Cell line	Reference
*H. sapiens*	X*	–	TMPyP4	100 µM	24, 36, and 48 h	HeLa S3	(Grand et al., 2002)
*H. sapiens*	V*	Custom algorithm, G_3_N_1–7_G_3_N_1–7_G_3_N_1–7_G_3_	TMPyP4	100 µM	24 and 48 h	HeLa S3	(Verma et al., 2008)
*H. sapiens*	X	–	TMPyP4	100 µM	48 h	K562	(Mikami-Terao et al., 2008)
*H. sapiens*	V	Custom algorithm, G_3_N_1–7_G_3_N_1–7_G_3_N_1–7_G_3_	TMPyP4	100 µM	48 h	HeLa S3, A549	(Verma et al., 2009)
*H. sapiens*	V	Custom algorithm, G_3_N_1–7_G_3_N_1–7_G_3_N_1–7_G_3_	PhenDC3, 360A	10 µM	48 h	HeLa S3	(Halder et al., 2012b)
List of G4 cis regulatory studies in bacteria							
Organism	Computational prediction	Prediction algorithm		Individual gene reporter assay	Transcriptome/differential expression analysis		Reference
*Escherichia coli*	V	Custom algorithm, G_2–5_N_1–5_G_2–5_N_1–5_G_2–5_N_1–5_G_2–5_		X	X		(Rawal et al., 2006)
*Deinococcus radiodurans*	V	Custom algorithm and QGRS Mapper, G_2–5_N_1–5_G_2–5_N_1–5_G_2–5_N_1–5_G_2–5_		V	X		(Kota et al., 2015)
*Mycobacterium tuberculosis*	V	Custom algorithm, _G2–5_N_7/11/15_G_2–5_N_7/11/15_G_2–5_N_7/11/15_G_2–5_		X	X		(Perrone et al., 2017)
*Xanthomonas* sp., and *Nostoc* sp.	V	ProQuad Database		X	X		(Rehm et al., 2015)
Domain bacteria (∼1500 genomes)	V	G4Hunter; Default parameters		X	X		(Bartas et al., 2019)
*E. coli*	V	Quadparser, G_≥3_N_1–7_ G_≥3_N_1–7_ G_≥3_N_1–7_G_≥3_		X	X		(Kaplan et al., 2016)
*E. coli*	V	ProQuad, G_2–5_N_1–5_G_2–5_N_1–5_G_2–5_N_1–5_G_2–5_		V	X		(Holder and Hartig, 2014)
*Streptococcus pneumoniae*	V	Custom algorithm, QGRS Mapper, PQSFinder, G_≥3_N_0–10_G_≥3_N_0–10_ G_≥3_N_0–10_G_≥3_		V	X		(Mishra et al., 2019)
*Rhodobacter*, *Trypanosoma*, *Plasmodium*, *E. coli*, *Leishmania*	V	Custom algorithm, G_3_N_1–12_G_3_N_1–12_G_3_N_1–12_G_3_ and G_2_N_1–12_G_2_N_1–12_G_2_N_1–12_G_2_		X	X		(Marsico et al., 2019)
Deinococcales and Thermales	V	Quadparser, G_3_N_1–12_G_3_N_1–12_G_3_N_1–12_G_3_ and G_2_N_1–12_G_2_N_1–12_G_2_N_1–12_G_2_		X	X		(Ding et al., 2018)
List of G4 cis-regulatory studies in viruses							
Organism	Computational prediction	Prediction algorithm		Individual gene reporter assay	Transcriptome/differential expression analysis		Reference
Human herpesvirus	V	Quadparser, G_3_N_1–7_ G_3_N_1–7_ G_3_N_1–7_G_3_		V	X		(Biswas et al., 2016)
Hepatitis B virus	V	Quadparser, G_3_N_1–7_ G_3_N_1–7_ G_3_N_1–7_G_3_		V	X		(Biswas et al., 2017)
Human cytomegalovirus	V	Custom script, G_3–6_N_1–7_G_3–6_N_1–7_G_3–6_N_1–7_G_3–6_		V	X		(Ravichandran et al., 2018)
Alphaherpesviruses	V	QGRS Mapper, Quadbase G_2_N_1–7_G_2_N_1–7_G_2_N_1–7_G_2_, G_3_N_1–12_G_3_N_1–12_G_3_N_1–12_G_3_		V	X		(Frasson et al., 2019)

“X” indicates “absent,” and “V” indicates “present.”

A similar study on the effect of gene expression by TMPyP4 in the K562 cell line showed that only 33 genes were upregulated and 54 genes were downregulated and proposed that TMPyP4 might act by repressing *CMYC* and activating MAPK family kinases (Mikami-Terao et al., 2008). The same group observed similar effects using retinoblastoma cell lines in response to TMPyP4 and demonstrated that the induction of p53 and activation of MAPK kinases could contribute to the antitumor effects of TMPyP4. However, in both studies, they could only speculate that the G4 stabilization by TMPyP4 could affect the regulation of differentially expressed genes. In addition, they also observed telomere shortening in both K562 and retinoblastoma cell lines where G4 stabilization prevents telomerase from binding to the 3′ end of the telomere and maintaining telomere length. So, it is difficult to predict whether the effect of G4 is by gene regulation or telomere shortening. TMPyP4 was developed to target telomeric G4s (Haq et al., 1999), but the transcriptome-wide study showed nonspecific activities, underscoring the need for further genome-wide studies in cells.

Another study compared the effect of bisquinolinium drugs 360A and PhenDC3 on gene expression in Hela S3 cell lines and showed that 1157 genes were downregulated and 1529 upregulated in PhenDC3. In the case of 360A, only 249 downregulated and 401 upregulated genes were observed (Halder et al., 2012b). This clearly indicates that although the small molecules were developed as G4-binding ligands, they show significant nonspecific effects, which need to be explored further. In addition, the mechanisms that dynamically control G4 formation are yet to be understood, so combined genome and transcriptome-wide mechanistic and functional analysis is required to unravel the mysteries of gene regulation by G4 stabilization.

### Studies on *Cis*-Regulatory G4s in Bacteria

The bacterial genome is considerably simpler than the eukaryotic genome due to the absence of the complex organization that is found in the human genome. However, various computational studies and individual promoter region analysis have shown that the genomes of several bacteria contain G4-forming sequences (**Table 1**). Genome-wide prediction has identified G4-forming sequences in the genomes of *Escherichia coli* (Rawal et al., 2006), *Deinococcus radiodurans* (Kota et al., 2015), *Mycobacterium tuberculosis* (Perrone et al., 2017), *Xanthomonas* sp., and *Nostoc* sp. (Rehm et al., 2015). These studies also showed that the G4-forming sequences were predominantly restricted to regulatory regions such as promoters (Rawal et al., 2006). In each study, individual luciferase assays carried out on selected promoter regions showed variable responses to the addition of G4 ligands. For example, in the case of *D.* radiodurans, some promoters showed higher activity when the bacterium was treated with NMM-IX, whereas others showed diminished activity, although all promoters contained G4-forming sequences (Kota et al., 2015). This lack of correlation exhibited in promoter luciferase assays and whole transcriptome studies indicates that the landscape of gene regulation by G4 is more complex than expected even in prokaryotes, despite the simple organization of their genome.

In the case of *E. coli*, a systematic study on the effect of the location of G4 relative to the transcription start site (TSS) was performed in the genome using reporter assays where the G4-forming sequences were cloned according to their genomic locations into pQE luciferase reporter plasmids (Holder and Hartig, 2014). The results revealed that G4 formation in the 5′ UTR significantly affected reporter gene expression, but the 3′ UTR G4s had a negligible effect on gene expression. It was also observed that the G4 sequences within 20 bp downstream of the TSS showed maximum upregulation or downregulation depending on whether the G4 was formed on the antisense or sense strand, respectively. Interestingly, there was no effect of NMM-IX or other G4-stabilizing ligands on G4-mediated gene regulation (Holder and Hartig, 2014), indicating that more studies are required to explain gene regulation by G4s in *E. coli*.

In a recent study on G4 sequences in the *E. coli* genome, the predicted G4-forming regions were aligned using ClustalW to identify repetitive sequence motifs (Kaplan et al., 2016). This was based on the idea that similar sequences will have similar regulatory roles. In this analysis, only 52 sequences matched their stringent schema with G-tract length 1 to 3 and loop length 1 to 7. They further classified these into two groups of well-aligned sequence and performed reporter assays using the representative sequence. Interestingly, all the sequences were within the regulatory regions flanking the open reading frame, and the group was able to identify two sequence motifs conserved in several bacteria. However, the functional impact of these sequences needs to be investigated.

The recent interest in studies on bacterial G4s has necessitated the formulation of a streamlined approach to facilitate interpretation of the roles of G4s in gene regulation. Since the bacterial system has been extensively studied as a model organism, it will be interesting to combine both high-throughput and individualistic approaches to gain a comprehensive picture of bacterial G4s.

### Studies on *Cis*-Regulatory G4s in Viruses

Viruses provide an exciting platform for studying the impact of G4s at the genomic and transcriptomic level due to their small genome size. Some viral DNA can be chromatinized as in eukaryotes, and the genetic materials of some DNA and RNA viruses can be integrated into the human genome and affected by the same parameters as human genomic DNA. Therefore, they present an ideal platform for studies that especially focus on the holistic effects of G4-binding ligands especially to understand G4-mediated gene regulation.

Genome-wide computational analyses of G4s in several viral genomes have revealed that DNA viruses had a higher number of G4s per 1 kb compared to RNA viruses (Lavezzo et al., 2018). Many computational and individual reporter studies were performed in a number of viruses to evaluate the *cis*-regulatory effects of G4s (**Table 1**). One of the first systematic genome-wide studies on G4s in viruses was performed in human herpesvirus genomes (Biswas et al., 2016). Preliminary computational analysis of G4s present in regulatory regions of the herpesvirus genome revealed their prevalence in regulatory and long terminal repeat regions. Among regulatory regions, immediate-early genes showed higher densities of G4-forming sequences when compared with early or late gene promoters. Overall, alpha-herpesviruses such as herpes simplex virus-1 (HSV-1) and varicella-zoster virus genomes had a higher G4-density than did human and mouse genomes, indicating that G4 formation in these viruses would have more impact on gene regulation. In this study, the authors considered G4-forming sequences found in only three genes, namely, UL2 and UL24 of HSV-1 and K15 of Kaposi’s sarcoma-associated herpesvirus, for further experimental study. It was observed that G4s could suppress gene expression in the presence of the G4-stabilizing ligands BRACO19 and TMPyP4. However, since several G4-forming sequences were predicted, the questions of how many G4-forming sequences actually form G4s and how many G4s regulate viral gene expression still need to be explored.

In lieu of this important question, a recent study systematically checked the effect of G4s on gene regulation in all genes of the human cytomegalovirus, which belongs to the beta-herpesvirus subfamily (Ravichandran et al., 2018). Unlike previous reports that only tested a few G4s, the genome-wide analysis for all conventional, long-loop, and bulged-G4 schema identified 36 G4-forming sequences associated with 20 viral genes including all immediate-early, early, and late genes. Most of these sequences formed G4s *in vitro*, and their stability could be further increased by NMM-IX treatment. The cell-based assays using reporter constructs with promoters containing G4s indicated that out of 20 genes only 9 were suppressed effectively by G4-stabilizing ligand NMM-IX. This is interesting because while all tested genes contained G4-forming sequences (evidenced by *in vitro* assays) in their promoter regions, only half of these genes were affected by the ligand. Therefore, it was proposed that there exists a context-dependent mechanism by which G4s influence viral genes. It is also possible that other factors that are involved in controlling gene expression, such as the binding of human transcription factors, are implicated in G4 activity as shown earlier for HPV (Carson and Khan, 2006). This proves to be an exciting field for further study and can facilitate the construction of G4-mediated regulatory networks. This also shows that although many promoters might contain G4s that can affect gene expression when tested individually, genome-wide studies are also important in studying the collective impact of G4 stabilization.

## Future Perspectives

The role of G4s in the genomes of prokaryotes and eukaryotes is an exciting area of research because of the plethora of reports showing the influence of G4s on specific gene expression. However, there is an absence of reconciliation between studies on individual G4s and high-throughput genome and transcriptome-wide studies, especially for *cis*-regulatory G4s. With the advent of next-generation sequencing technologies and high-throughput reporter assays, it should be possible to construct complex G4 networks with the ability to incorporate computational and experimental analysis and present a combined view of G4-mediated regulation. For example, the computational G4 prediction and individual gene reporter assays can be compared with high-throughput differential expression studies to identify candidates that show the maximum effect. The regions can be compared with Chromatin Immuno Precipitation (ChIP) studies to correlate with transcription factor binding sites or chromatin binding sites. Construction of a central repository to store the results of functional analysis from various publications will also facilitate the comparison of data and provide a holistic picture of gene regulation by G4s.

## Author Contributions

SR, J-HA, and KK wrote the manuscript.

## Funding

This work was supported by National Research Foundation of Korea funded by the Ministry of Science and ICT (2019R1A2C2006676 to J-HA and 2019R1A2C2089148 to KK).

## Conflict of Interest

The authors declare that the research was conducted in the absence of any commercial or financial relationships that could be construed as a potential conflict of interest.

## References

[B1] Abou Assi H. Garavis M. Gonzalez C. Damha M. J. (2018). i-Motif DNA: structural features and significance to cell biology. Nucleic Acids Res. 46, 8038–8056. 10.1093/nar/gky735 30124962 PMC6144788

[B2] Ashman L. K. Griffith R. (2013). Therapeutic targeting of c-KIT in cancer. Expert Opin Investig. Drugs 22, 103–115. 10.1517/13543784.2013.740010 23127174

[B3] Bartas M. Cutova M. Brazda V. Kaura P. Stastny J. Kolomaznik J. (2019). The presence and localization of G-Quadruplex forming sequences in the domain of bacteria. Molecules 24. 10.3390/molecules24091711 PMC6539912 31052562

[B4] Bhattacharyya D. Mirihana Arachchilage G. Basu S. (2016). Metal Cations in G-Quadruplex Folding and Stability. Front Chem. 4, 38. 10.3389/fchem.2016.00038 27668212 PMC5016522

[B5] Biswas B. Kandpal M. Jauhari U. K. Vivekanandan P. (2016). Genome-wide analysis of G-quadruplexes in herpesvirus genomes. BMC Genomics 17, 949. 10.1186/s12864-016-3282-1 27871228 PMC5117502

[B6] Biswas B. Kandpal M. Vivekanandan P. (2017). A G-quadruplex motif in an envelope gene promoter regulates transcription and virion secretion in HBV genotype B. Nucleic Acids Res. 45, 11268–11280. 10.1093/nar/gkx823 28981800 PMC5737607

[B7] Bothe J. R. Lowenhaupt K. Al-Hashimi H. M. (2011). Sequence-specific B-DNA flexibility modulates Z-DNA formation. J. Am. Chem. Soc. 133, 2016–2018. 10.1021/ja1073068 21275369 PMC3319140

[B8] Brazda V. Haronikova L. Liao J. C. Fojta M. (2014). DNA and RNA quadruplex-binding proteins. Int. J. Mol. Sci. 15, 17493–17517. 10.3390/ijms151017493 25268620 PMC4227175

[B9] Brazda V. Laister R. C. Jagelska E. B. Arrowsmith C. (2011). Cruciform structures are a common DNA feature important for regulating biological processes. BMC Mol. Biol. 12, 33. 10.1186/1471-2199-12-33 21816114 PMC3176155

[B10] Carson A. Khan S. A. (2006). Characterization of transcription factor binding to human papillomavirus type 16 DNA during cellular differentiation. J. Virol 80, 4356–4362. 10.1128/JVI.80.9.4356-4362.2006 16611894 PMC1472023

[B11] Cer R. Z. Bruce K. H. Mudunuri U. S. Yi M. Volfovsky N. Luke B. T. (2011). Non-B DB: a database of predicted non-B DNA-forming motifs in mammalian genomes. Nucleic Acids Res. 39, D383–D391. 10.1093/nar/gkq1170 PMC3013731 21097885

[B12] Chambers V. S. Marsico G. Boutell J. M. Di Antonio M. Smith G. P. Balasubramanian S. (2015). High-throughput sequencing of DNA G-quadruplex structures in the human genome. Nat. Biotechnol. 33, 877–881. 10.1038/nbt.3295 26192317

[B13] Cogoi S. Xodo L. E. (2006). G-quadruplex formation within the promoter of the KRAS proto-oncogene and its effect on transcription. Nucleic Acids Res. 34, 2536–2549. 10.1093/nar/gkl286 16687659 PMC1459413

[B14] Ding Y. Fleming A. M. Burrows C. J. (2018). Case studies on potential G-quadruplex-forming sequences from the bacterial orders Deinococcales and Thermales derived from a survey of published genomes. Sci. Rep. 8, 15679. 10.1038/s41598-018-33944-4 30356061 PMC6200779

[B15] Eddy J. Maizels N. (2006). Gene function correlates with potential for G4 DNA formation in the human genome. Nucleic Acids Res. 34, 3887–3896. 10.1093/nar/gkl529 16914419 PMC1557811

[B16] Fay M. M. Lyons S. M. Ivanov P. (2017). RNA G-Quadruplexes in Biology: Principles and Molecular Mechanisms. J Mol. Biol 429, 2127–2147. 10.1016/j.jmb.2017.05.017 28554731 PMC5603239

[B17] Fedoroff O. Y. Rangan A. Chemeris V. V. Hurley L. H. (2000). Cationic porphyrins promote the formation of i-motif DNA and bind peripherally by a nonintercalative mechanism. Biochemistry 39, 15083–15090. 10.1021/bi001528j 11106486

[B18] Frank-Kamenetskii M. D. Mirkin S. M. (1995). Triplex DNA structures. Annu. Rev. Biochem. 64, 65–95. 10.1146/annurev.bi.64.070195.000433 7574496

[B19] Frasson I. Nadai M. Richter S. N. (2019). Conserved G-Quadruplexes Regulate the Immediate Early Promoters of Human Alphaherpesviruses. Molecules 24. 10.3390/molecules24132375 PMC6651000 31252527

[B20] Grand C. L. Han H. Munoz R. M. Weitman S. Von Hoff D. D. Hurley L. H. (2002). The cationic porphyrin TMPyP4 down-regulates c-MYC and human telomerase reverse transcriptase expression and inhibits tumor growth in vivo. Mol. Cancer Ther. 1, 565–573. 12479216

[B21] Halder K. Benzler M. Hartig J. S. (2012a). Reporter assays for studying quadruplex nucleic acids. Methods 57, 115–121. 10.1016/j.ymeth.2012.02.005 22388183

[B22] Halder R. Riou J. F. Teulade-Fichou M. P. Frickey T. Hartig J. S. (2012b). Bisquinolinium compounds induce quadruplex-specific transcriptome changes in HeLa S3 cell lines. BMC Res. Notes 5, 138. 10.1186/1756-0500-5-138 22414013 PMC3375199

[B23] Hale T. K. Norris G. E. Jameson G. B. Filichev V. V. (2014). Helicases, G4-DNAs, and drug design. ChemMedChem 9, 2031–2034. 10.1002/cmdc.201402068 24825788

[B24] Hansel-Hertsch R. Beraldi D. Lensing S. V. Marsico G. Zyner K. Parry A. (2016). G-quadruplex structures mark human regulatory chromatin. Nat. Genet. 48, 1267–1272. 10.1038/ng.3662 27618450

[B25] Haq I. Trent J. O. Chowdhry B. Z. Jenkins T. C. (1999). Intercalative G-Tetraplex stabilization of telomeric DNA by a cationic porphyrin. J. Am. Chem. Soc. 121, 1768–1779.

[B26] Hazel P. Huppert J. Balasubramanian S. Neidle S. (2004). Loop-length-dependent folding of G-quadruplexes. J. Am. Chem. Soc. 126, 16405–16415. 10.1021/ja045154j 15600342

[B27] Holder I. T. Hartig J. S. (2014). A matter of location: influence of G-quadruplexes on Escherichia coli gene expression. Chem. Biol. 21, 1511–1521. 10.1016/j.chembiol.2014.09.014 25459072

[B28] Huppert J. L. Balasubramanian S. (2005). Prevalence of quadruplexes in the human genome. Nucleic Acids Res. 33, 2908–2916. 10.1093/nar/gki609 15914667 PMC1140081

[B29] Huppert J. L. Balasubramanian S. (2007). G-quadruplexes in promoters throughout the human genome. Nucleic Acids Res. 35, 406–413. 10.1093/nar/gkl1057 17169996 PMC1802602

[B30] Huppert J. L. Bugaut A. Kumari S. Balasubramanian S. (2008). G-quadruplexes: the beginning and end of UTRs. Nucleic Acids Res. 36, 6260–6268. 10.1093/nar/gkn511 18832370 PMC2577360

[B31] Kaplan O. I. Berber B. Hekim N. Doluca O. (2016). G-quadruplex prediction in E. coli genome reveals a conserved putative G-quadruplex-Hairpin-Duplex switch. Nucleic Acids Res. 44, 9083–9095. 10.1093/nar/gkw769 27596596 PMC5100583

[B32] Kim D. Hur J. Han J. H. Ha S. C. Shin D. Lee S. (2018). Sequence preference and structural heterogeneity of BZ junctions. Nucleic Acids Res. 46, 10504–10513. 10.1093/nar/gky784 30184200 PMC6212838

[B33] Kim D. Reddy S. Kim D. Y. Rich A. Lee S. Kim K. K. (2009). Base extrusion is found at helical junctions between right- and left-handed forms of DNA and RNA. Nucleic Acids Res. 37, 4353–4359. 10.1093/nar/gkp364 19465399 PMC2715235

[B34] Kota S. Dhamodharan V. Pradeepkumar P. I. Misra H. S. (2015). G-quadruplex forming structural motifs in the genome of Deinococcus radiodurans and their regulatory roles in promoter functions. Appl. Microbiol. Biotechnol 99, 9761–9769. 10.1007/s00253-015-6808-6 26201493

[B35] Kreig A. Calvert J. Sanoica J. Cullum E. Tipanna R. Myong S. (2015). G-quadruplex formation in double strand DNA probed by NMM and CV fluorescence. Nucleic Acids Res. 43, 7961–7970. 10.1093/nar/gkv749 26202971 PMC4652765

[B36] Kwok C. K. Marsico G. Sahakyan A. B. Chambers V. S. Balasubramanian S. (2016). rG4-seq reveals widespread formation of G-quadruplex structures in the human transcriptome. Nat. Methods. 13, 841–844. 10.1038/nmeth.3965 27571552

[B37] Kwok C. K. Merrick C. J. (2017). G-Quadruplexes: Prediction, Characterization, and Biological Application. Trends Biotechnol. 35, 997–1013. 10.1016/j.tibtech.2017.06.012 28755976

[B38] Lavezzo E. Berselli M. Frasson I. Perrone R. Palu G. Brazzale A. R. (2018). G-quadruplex forming sequences in the genome of all known human viruses: A comprehensive guide. PLoS Comput. Biol 14, e1006675. 10.1371/journal.pcbi.1006675 PMC6307822 30543627

[B39] Le V. H. Nagesh N. Lewis E. A. (2013). Bcl-2 promoter sequence G-quadruplex interactions with three planar and non-planar cationic porphyrins: TMPyP4, TMPyP3, and TMPyP2 . PLoS One 8, e72462. 10.1371/journal.pone.0072462 PMC3748076 23977303

[B40] Leach D. R. (1994). Long DNA palindromes, cruciform structures, genetic instability and secondary structure repair. Bioessays 16, 893–900. 10.1002/bies.950161207 7840768

[B41] Marsico G. Chambers V. S. Sahakyan A. B. Mccauley P. Boutell J. M. Antonio M. D. (2019). Whole genome experimental maps of DNA G-quadruplexes in multiple species. Nucleic Acids Res. 47, 3862–3874. 10.1093/nar/gkz179 30892612 PMC6486626

[B42] Masoud S. S. Nagasawa K. (2018). i-Motif-Binding Ligands and Their Effects on the Structure and Biological Functions of i-Motif. Chem. Pharm. Bull 66, 1091–1103. 10.1248/cpb.c18-00720 30504626

[B43] Membrino A. Cogoi S. Pedersen E. B. Xodo L. E. (2011). G4-DNA formation in the HRAS promoter and rational design of decoy oligonucleotides for cancer therapy. PLoS One 6, e24421. 10.1371/journal.pone.0024421 PMC3169596 21931711

[B44] Mikami-Terao Y. Akiyama M. Yuza Y. Yanagisawa T. Yamada O. Yamada H. (2008). Antitumor activity of G-quadruplex-interactive agent TMPyP4 in K562 leukemic cells. Cancer Lett. 261, 226–234. 10.1016/j.canlet.2007.11.017 18096315

[B45] Mishra S. K. Jain N. Shankar U. Tawani A. Sharma T. K. Kumar A. (2019). Characterization of highly conserved G-quadruplex motifs as potential drug targets in Streptococcus pneumoniae. Sci. Rep. 9, 1791. 10.1038/s41598-018-38400-x 30741996 PMC6370756

[B46] Morris M. J. Wingate K. L. Silwal J. Leeper T. C. Basu S. (2012). The porphyrin TmPyP4 unfolds the extremely stable G-quadruplex in MT3-MMP mRNA and alleviates its repressive effect to enhance translation in eukaryotic cells. Nucleic Acids Res. 40, 4137–4145. 10.1093/nar/gkr1308 22266651 PMC3351169

[B47] Nagesh N. Sharma V. K. Ganesh Kumar A. Lewis E. A. (2010). Effect of ionic strength on porphyrin drugs interaction with quadruplex DNA formed by the promoter region of C-myc and Bcl2 oncogenes. J Nucleic Acids 2010. 10.4061/2010/146418 PMC2911617 20700417

[B48] Ofer N. Weisman-Shomer P. Shklover J. Fry M. (2009). The quadruplex r(CGG)n destabilizing cationic porphyrin TMPyP4 cooperates with hnRNPs to increase the translation efficiency of fragile X premutation mRNA. Nucleic Acids Res. 37, 2712–2722. 10.1093/nar/gkp130 19273535 PMC2677883

[B49] Pagano A. Iaccarino N. Abdelhamid M.a.S. Brancaccio D. Garzarella E. U. Di Porzio A. (2018). Common G-Quadruplex Binding Agents Found to Interact With i-Motif-Forming DNA: Unexpected Multi-Target-Directed Compounds. Front Chem. 6, 281. 10.3389/fchem.2018.00281 30137743 PMC6066642

[B50] Parveen N. Shamim A. Cho S. Kim K. K. (2019). Computational approaches to predict the noncanonical DNAs. Current Bioinf. 14. 10.2174/1574893614666190126143438

[B51] Perrone R. Lavezzo E. Riello E. Manganelli R. Palu G. Toppo S. (2017). Mapping and characterization of G-quadruplexes in Mycobacterium tuberculosis gene promoter regions. Sci. Rep. 7, 5743. 10.1038/s41598-017-05867-z 28720801 PMC5515968

[B52] Ravichandran S. Kim Y. E. Bansal V. Ghosh A. Hur J. Subramani V. K. (2018). Genome-wide analysis of regulatory G-quadruplexes affecting gene expression in human cytomegalovirus. PLoS Pathog 14, e1007334. 10.1371/journal.ppat.1007334 PMC6179306 30265731

[B53] Rawal P. Kummarasetti V. B. Ravindran J. Kumar N. Halder K. Sharma R. (2006). Genome-wide prediction of G4 DNA as regulatory motifs: role in Escherichia coli global regulation. Genome Res. 16, 644–655. 10.1101/gr.4508806 16651665 PMC1457047

[B54] Rehm C. Wurmthaler L. A. Li Y. Frickey T. Hartig J. S. (2015). Investigation of a Quadruplex-Forming Repeat Sequence Highly Enriched in Xanthomonas and Nostoc sp. PLoS One 10, e0144275. 10.1371/journal.pone.0144275 PMC4692102 26695179

[B55] Sannohe Y. Sugiyama H. (2010). Overview of formation of G-quadruplex structures. Curr. Protoc. Nucleic Acid Chem. 12 11–12 17. 10.1002/0471142700.nc1702s40 20201027

[B56] Siddiqui-Jain A. Grand C. L. Bearss D. J. Hurley L. H. (2002). Direct evidence for a G-quadruplex in a promoter region and its targeting with a small molecule to repress c-MYC transcription. Proc Natl. Acad. Sci. U. S. A. 99, 11593–11598. 10.1073/pnas.182256799 12195017 PMC129314

[B57] Simone R. Fratta P. Neidle S. Parkinson G. N. Isaacs A. M. (2015). G-quadruplexes: Emerging roles in neurodegenerative diseases and the non-coding transcriptome. FEBS Lett. 589, 1653–1668. 10.1016/j.febslet.2015.05.003 25979174

[B58] Tian T. Chen Y. Q. Wang S. R. Zhou X. (2018). G-Quadruplex: A Regulator of Gene Expression and Its Chemical Targeting. Chem 4, 1314–1344. 10.1016/j.chempr.2018.02.014

[B59] Verma A. Halder K. Halder R. Yadav V. K. Rawal P. Thakur R. K. (2008). Genome-wide computational and expression analyses reveal G-quadruplex DNA motifs as conserved cis-regulatory elements in human and related species. Journal of Medicinal Chemistry 51, 5641–5649. 10.1021/jm800448a 18767830

[B60] Verma A. Yadav V. K. Basundra R. Kumar A. Chowdhury S. (2009). Evidence of genome-wide G4 DNA-mediated gene expression in human cancer cells. Nucleic Acids Res. 37, 4194–4204. 10.1093/nar/gkn1076 19211664 PMC2715224

[B61] Wu Y. Brosh R. M. Jr. (2010). G-quadruplex nucleic acids and human disease. FEBS J 277, 3470–3488. 10.1111/j.1742-4658.2010.07760.x 20670277 PMC2923685

[B62] Yadav V. K. Abraham J. K. Mani P. Kulshrestha R. Chowdhury S. (2008). QuadBase: genome-wide database of G4 DNA–occurrence and conservation in human, chimpanzee, mouse and rat promoters and 146 microbes. Nucleic Acids Res. 36, D381–D385. 10.1093/nar/gkm781 PMC2238983 17962308

[B63] Yang F. M. Sun X. Wang L. X. Li Q. Guan A. J. Shen G. (2017). Selective recognition of c-myc promoter G-quadruplex and down-regulation of oncogene c-myc transcription in human cancer cells by 3,8a-disubstituted indolizinone. Rsc. Adv. 7, 51965–51969. 10.1039/C7RA09870G

[B64] Yang S. Y. Lejault P. Chevrier S. Boidot R. Robertson A. G. Wong J. M. Y. (2018). Transcriptome-wide identification of transient RNA G-quadruplexes in human cells. Nat. Commun. 9, 4730. 10.1038/s41467-018-07224-8 30413703 PMC6226477

[B65] Zaccaria F. Paragi G. Fonseca Guerra C. (2016). The role of alkali metal cations in the stabilization of guanine quadruplexes: why K(+) is the best. Phys. Chem. Chem. Phys. 18, 20895–20904. 10.1039/C6CP01030J 27185388

[B66] Zamiri B. Reddy K. Macgregor R. B. Jr., and Pearson C. E. (2014). TMPyP4 porphyrin distorts RNA G-quadruplex structures of the disease-associated r(GGGGCC)n repeat of the C9orf72 gene and blocks interaction of RNA-binding proteins. J Biol. Chem. 289, 4653–4659. 10.1074/jbc.C113.502336 24371143 PMC3931028

